# A Cryophyte Transcription Factor, CbABF1, Confers Freezing, and Drought Tolerance in Tobacco

**DOI:** 10.3389/fpls.2019.00699

**Published:** 2019-05-31

**Authors:** Xiule Yue, Guoyan Zhang, Zhen Zhao, Jinli Yue, Xiaohong Pu, Mengjun Sui, Yi Zhan, Yulan Shi, Zhenyu Wang, Guanghua Meng, Zhixing Zhao, Lizhe An

**Affiliations:** ^1^Ministry of Education Key Laboratory of Cell Activities and Stress Adaptations, School of Life Sciences, Lanzhou University, Lanzhou, China; ^2^Cuiying Honors College, Lanzhou University, Lanzhou, China; ^3^Extreme Stress Resistance and Biotechnology Laboratory, Northwest Institute of Eco-Environment and Resources, Chinese Academy of Sciences, Lanzhou, China; ^4^Institute of Tropical Agriculture and Forestry, Hainan University, Haikou, China

**Keywords:** ABF, abiotic stress, cryophyte, *Chorispora bungeana*, cold stress, drought tolerance

## Abstract

Abscisic acid responsive element binding factors (ABFs) play crucial roles in plant responses to abiotic stress. However, little is known about the roles of ABFs in alpine subnival plants, which can survive under extreme environmental conditions. Here, we cloned and characterized an *ABF1* homolog, *CbABF1*, from the alpine subnival plant *Chorispora bungeana*. Expression of *CbABF1* was induced by cold, drought, and abscisic acid. Subcellular localization analysis revealed that CbABF1 was located in the nucleus. Further, CbABF1 had transactivation activity, which was dependent on the N-terminal region containing 89 residues. A Snf1-related protein kinase, CbSnRK2.6, interacted with CbABF1 in yeast two-hybrid analysis and bimolecular fluorescence complementation assays. Transient expression assay revealed that CbSnRK2.6 enhanced the transactivation of CbABF1 on ABRE *cis*-element. We further found that heterologous expression of *CbABF1* in tobacco improved plant tolerance to freezing and drought stress, in which the survival rates of the transgenic plants increased around 40 and 60%, respectively, compared with wild-type plants. Moreover, the transgenic plants accumulated less reactive oxygen species, accompanied by high activities of antioxidant enzymes and elevated expression of stress-responsive genes. Our results thus suggest that CbABF1 is a transcription factor that plays an important role in cold and drought tolerance and is a candidate gene in molecular breeding of stress-tolerant crops.

## Introduction

Plant growth, development, and productivity can be adversely affected by environmental stresses including cold, drought and high salinity. To respond and adapt to these abiotic stresses, plants undergo changes at the molecular, cellular, and physiological levels (Kreps et al., 2002; Chen and Zhu, 2004; Shinozaki and Yamaguchi-Shinozaki, 2007; Cutler et al., 2010). Various transcription factors act as major mediators of plant abiotic-stress tolerance by binding to specific *cis*-acting elements in the promoters of stress-responsive genes, thus enabling the plant to withstand adverse environmental conditions (Cutler et al., 2010; Song et al., 2016). Among these transcription factors, basic leucine zipper (bZIP) is a large family that harbors a bZIP domain containing a basic region and a leucine zipper (Jakoby et al., 2002); many of these factors have important functions in plant stress-response and hormone signal transduction (Kim, 2005). A subfamily of bZIP transcription factors bind to the ABA response element (ABRE; PyACGTGG/TC) to activate downstream genes in response to abiotic stress, and accordingly are designated as ABRE-binding factors (ABFs) or ABRE-binding proteins (AREBs) (Choi et al., 2000; Uno et al., 2000). Several ABF/AREB transcription factors have been characterized in *Arabidopsis thaliana* (Yoshida et al., 2015; Wang et al., 2018), rice (*Oryza sativa*) (Xiang et al., 2008; Amir Hossain et al., 2010), wheat (*Triticum aestivum*) (Johnson et al., 2008), soybean (*Glycine max*) (Gao et al., 2011), and tomato (*Solanum lycopersicum*) (Orellana et al., 2010).

*Arabidopsis* encodes nine ABF/AREB proteins that harbor one conserved C-terminal domain and three conserved N-terminal domains in addition to a bZIP domain in which the leucine zipper region mediates homo- and hetero-dimerization of the transcription factors (Uno et al., 2000; Yoshida et al., 2010, 2015). Among the nine ABF/AREB genes, three (*ABF2*/*AREB1*, *ABF3*, and *ABF4*/*AREB2*) are substantially upregulated by osmotic stress and ABA treatment in vegetative tissues (Kang, 2002; Fujita et al., 2005; Yoshida et al., 2010). *ABF1* is induced by high salinity, dehydration, and ABA treatment, but to levels that are markedly lower than those of above three genes. *ABF1* and *ABF4* are also induced by cold stress, although the induction level of *ABF4* is relatively low (Yoshida et al., 2015; Fernando et al., 2018). In summary, four *ABF*/*AREB* genes are induced by environmental stresses, with different ABF/AREBs appearing to play diverse roles in response to different stresses.

Although ABF/AREB transcription factors are induced by abiotic stress, they require ABA for full activation, and their activities are regulated by ABA-dependent phosphorylation. As endogenous ABA levels increase in response to abiotic stress (Zhu, 2002; Yamaguchi-Shinozaki and Shinozaki, 2006), ABA binds PYRABACTIN RESISTANCE 1/PYRABACTIN RESISTANCE 1-LIKE/REGULATORY COMPONENT OF ABA RECEPTOR (PYR/PYL/RCAR) proteins, which in turn creates an interaction surface to recruit and inhibit TYPE 2C PROTEIN PHOSPHATASEs (PP2Cs), leading to the release and activation of SUCROSE NON-FERMENTING 1 (SNF1)-RELATED PROTEIN KINASE 2 (SnRK2/SRK2) proteins, which directly phosphorylate and positively control the ABF/AREB transcription factors (Fujita et al., 2009; Cutler et al., 2010; Fujita et al., 2013). Recently, Chen et al. (2018) demonstrated that *Arabidopsis* EL1-like (AEL) protein could suppress ABA responses by phosphorylating PYR/PYLs to promote their ubiquitination and degradation.

A number of downstream genes of ABF/AREBs have been described, including *RD29B*, *LEA*, *KAT1*, *ADH1*, and *RD20* (Fujita et al., 2013; Yoshida et al., 2015). In addition, genes that produce and scavenge reactive oxygen species (ROS) are reported to be regulated by ABFs to reduce oxidative damage in *Arabidopsis* and other species (Huang et al., 2010; Zhang et al., 2011). Furthermore, some studies have found improvement of plant stress resistance upon over-expression of transcription factors, such as *ZmABP9* (*Zea mays ABP9*) (Zhang et al., 2011), *AhAREB1* (*Arachis hypogaea AREB1*) (Li et al., 2013), and *GmbZIP1* (*G. max bZIP1*) (Gao et al., 2011). Transgenic *Arabidopsis* overexpressing ABF3 or ABF4 showed tolerance to high salinity and drought, with expression levels changed in a number of downstream genes (Kang, 2002; Kim et al., 2004; Fujita et al., 2005). Over-expression of rice *OsABF1*, *OsbZIP23*, or *OsbZIP72* resulted in enhanced tolerance to abiotic stress (Xiang et al., 2008; Lu et al., 2009; Amir Hossain et al., 2010). These findings point to over-expression of certain stress-responsive genes as a strategy to improve the tolerance of crops to abiotic stress.

**FIGURE 1:**
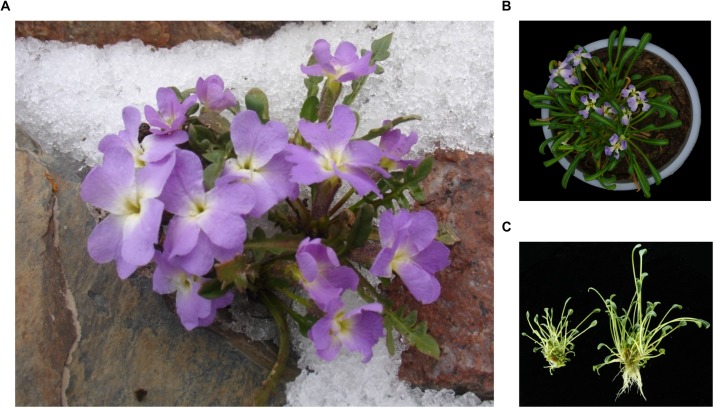
Morphology of *Chorispora bungeana*. **(A)** Flowering stage of plants in their natural habitat (rocky mountains in snow). **(B)** Plants flowering in a growth chamber. **(C)** Regenerated seedlings from tissue culture.

*Chorispora bungeana* (Brassicaceae) is a representative alpine subnival perennial herb that thrives even amid freezing temperatures and frequent temperature fluctuations (Figure 1). *C. bungeana* is distributed mainly beside Urumqi Glacier No. 1 at an altitude of 3800–3900 m near the Urumqi River in the Tianshan Mountains of Xinjiang province, China, with a monthly mean temperature of only 3–5°C in summer (June–August), with occasional snow or hail storms (Ye et al., 2005). *C. bungeana* exhibits no obvious stress-related morphological characteristics; thus, physiological or molecular mechanisms are believed to contribute to its adaptation to this extremely stressful environment. In previous studies, several stress-responsive genes from *C. bungeana* have been cloned and characterized, including *CbFAD3* (*C. bungeana ω-3 fatty acid desaturase*) (Shi et al., 2018), *CbPLD* (*C. bungeana phospholipase D*) (Yang et al., 2011), and *CbDRH* (*C. bungeana DEAD-box RNA helicase*) (Yang et al., 2014). Understanding the mechanisms of *C. bungeana* in adaptation to stress may inform efforts to improve the stress tolerance of crops.

Transcriptome profiles of cold-treated *C. bungeana* suggest that transcription factors play crucial roles in the rapid response and adaptation to environmental stresses (Zhao et al., 2012). However, little is known about the function of ABF/AREB transcription factors of this alpine subnival plant in response to stress or whether ABF/AREB genes from the subnival plant could be used to enhance stress tolerance in transgenic plants. In this study, *CbABF1* (*C. bungeana ABF1*), an *ABF1* homolog, was cloned and characterized. Our results indicate that *CbABF1* encodes a bZIP transcript factor that is induced by freezing and drought stress. We also reveal that CbABF1 interacts with CbSnRK2.6 (*C. bungeana* SnRK2.6), a Snf1-related protein kinase that may activate CbABF1 by phosphorylation. In addition, over-expression of *CbABF1* greatly enhanced freezing and drought tolerance in the transgenic plants, which accumulated lower levels of reactive oxygen species, accompanied by high activities of antioxidant enzymes and elevated expression of stress-responsive genes, suggesting that *CbABF1* is a strong candidate gene to improve the stress tolerance of crops in the future.

## Materials and Methods

### Plant Materials and Stress Treatments

Seeds of *C. bungeana* were collected from an ice-free cirque (43°05′N, 86°49′E, altitude 3800–3900 m.a.s.l.) near the Urumqi No. 1 Glacier in the source region of the Urumqi River in the Tianshan Mountains, Xinjiang Province, China. Embryogenic calli were produced from mature seeds and regenerated plants were obtained as described previously (Fu et al., 2006), with some modifications. Briefly, the hard seed coats were removed with sandpaper and seeds were rinsed in 70% ethanol for 30 s, surface sterilized in a 2% hypochlorite solution plus 0.01% Triton X-100 for 10 min and then washed five times with sterilized water. The cotyledons were cut in half and placed on solid MS medium supplemented with 1 mg/L 2,4-dichlorophenoxyacetic acid (2,4-D), 0.2 mg/L 6-benzyladenine (6-BA), 0.2 mg/L α-naphthalene acetic acid and 3% sucrose, and cultured at 20°C under a 16:8 (light: dark) photoperiod with light irradiance of 100 μmol m^-2^ s^-1^ and relative humidity 60%. Seedlings were regenerated from calli on solid MS medium containing 1 mg/L 6-BA and 3% sucrose in about 3 weeks. The seedlings were sub-cultured once per month. Regenerated plants from the second subculture were subjected treatment conditions when they were approximately 5 cm tall (2 weeks after subculture). The salinity (250 mM NaCl), freezing (-4°C), ABA (100 μM) treatments were over a time course of 0, 2, 4, 8, 12, 16, or 24 h. For the drought treatment, shoots of *C. bungeana* were placed on filter papers (150 mm × 150 mm) and dried for 0, 2, 4, 8, or 12 h. All samples were analyzed immediately or immersed rapidly in liquid nitrogen and stored at -80°C for subsequent analysis.

Tobacco (*Nicotiana tabacum*) plants were grown in a growth chamber (SANYO, MLR-351H) at 25°C under 16:8 (light:dark) with a light intensity of 100 μmol m^-2^ s^-1^ provided by cool-white fluorescent lights.

### Cloning and Bioinformatics Analysis of CbABF1

Based on the EST (GenBank: JK711564.1) from *C. bungeana*, RACE was performed to determine the 3′ and 5′ ends of *CbABF1* using the GeneRacer^TM^ RACE Ready cDNA Kit (Invitrogen, Carlsbad, CA, United States). The amplified fragments were cloned into the PMD18-T (Takara, Dalian, China) vector for sequencing. The full-length cDNA sequence of *CbABF1* was assembled from the 5′-RACE, 3′-RACE and the middle region sequences with SeqMan software. Multiple alignments of CbABF1 and other ABFs were performed with DNAMAN software. A phylogenetic tree was constructed with Mega 7.0 program by the neighbor-joining method. Bootstrap analysis was performed with 1,000 replicates, with values shown as percentages (Kumar et al., 2016). Domains of CbABF1 were predicted on a web-based protein database, SMART (Letunic and Bork, 2018).

### *CbABF1* Expression Analysis by Quantitative Reverse Transcription PCR (qRT-PCR)

Quantitative reverse transcription-PCR was performed to evaluate the expression levels of *CbABF1* in different treatments. Gene-specific primers (Supplementary Table S1) were designed by Primer Premier (v. 6.0) (PREMIER Biosoft International, Palo Alto, CA, United States). The primer specificities were confirmed by BLASTN with *C. bungeana* unigene transcripts (Zhao et al., 2012). The *C. bungeana ACTIN* gene (AY825362) was taken as an endogenous invariant control for normalization (Shi et al., 2018).

Total RNA was isolated and digested with DNase I by RNAprep pure Plant Kit (TianGen, Beijing, China). The cDNA synthesis was performed using PrimeScript^TM^ II 1st Strand cDNA Synthesis Kit (Takara). Each qRT-PCR reaction mixture was prepared according to the instructions of SYBR^®^ Green Supermix kit (Bio-Rad, Hercules, CA, United States). The PCR program was as follows: initial denaturation at 95°C for 3 min followed by amplification for 40 cycles at 95°C for 20 s, 57°C for 20 s, and 72°C for 30 s. Melting curve analysis was performed from 55 to 95°C with a heating rate of 0.1°C/s and continuous fluorescence measurements. After the reactions, threshold cycle (CT) values were obtained, and the relative transcript level (in fold changes) between the untreated (control) and treated plants was calculated using Pfaffl’s method (Pfaffl, 2001). All reactions were carried out in iCycler iQ^®^ multicolor real-time PCR Detection System (Bio-Rad). The fold change in the transcript levels was presented as the mean ± the standard deviation (SD) of three biological replicates. Amplification efficiencies for each PCR product were determined according to Bio-Rad real-time PCR application guide.

### Subcellular Localization of YFP Fusion Proteins

The protein-coding region of *CbABF1* was amplified and inserted into the pDONR/zeo vector (Invitrogen). Validated cDNA inserts were then sub-cloned into pEarleyGate 101 for C-terminal YFP fusion. The resulting CbABF1-YFP fusion constructs driven by the CaMV35S promoter were delivered into tobacco leaves by *Agrobacterium*-mediated transformation. Expression of fusion constructs was monitored after 24 h incubation in the dark by a confocal laser scanning microscope (Leica TCS SP8, Germany). DAPI was used to stain the nucleus.

### Transcriptional Activation Assay of CbABF1

The transactivation analysis was performed using Matchmaker Two-Hybrid System 3 according to the manufacturer’s instructions (Clontech, Mountain View, CA, United States). Full length and truncated protein-coding regions of *CbABF1* were amplified with specific primers harboring NdeI and SalI restriction sites (Supplementary Table S1, YH1 and YH393 for *CbABF1*, YH1, and YH89 for *CbABF1Δ1*, YH90, and YH393 for *CbABF1Δ2*). After double digestion, the resultant fragments were purified and sub-cloned into vector pGBKT7 which has a GAL4 DNA-binding domain to get fusion constructs (pGBKT7-CbABF1, pGBKT7-CbABF1Δ1, and pGBKT7-CbABF1Δ2). These constructs and empty pGBKT7 were separately co-transformed with pGADT7 into yeast strain AH109, and the transformants were cultivated at 30°C for 4 days on the SD/-Trp/-Leu or SD/-Trp/-Leu/-His medium supplemented with 35 mM 3-AT. Growth status evaluation and X-gal assay were performed to determine the transcriptional activation, as described in the manual.

### Yeast Two-Hybrid Assay

A yeast two-hybrid cDNA library from *C. bungeana* was constructed using Matchmaker^TM^ Library Construction and Screening Kits (Clontech, Mountain View, CA, United States). Briefly, total RNA from *C. bungeana* seedlings was isolated and digested with DNase I by RNAprep pure Plant Kit (TianGen, Beijing, China). After the first strand of cDNA was synthesized, double-stranded cDNA (ds cDNA) was generated and amplified by 20 cycles of long-distance PCR using Advantage 2 Polymerase Mix (Clontech). The purified double-stranded cDNA was inserted into a *Sma* I-linearized pGADT7-Rec vector (3 μg) with T4 DNA ligase (New England Biolabs, Ipswich, MA, United States). The constructs were then electro-transformed into *Escherichia coli* DH5α. The constructed cDNA library was evaluated for the number of independent clones and transformation efficiency.

For yeast two-hybrid assays, the bait construct pGBKT7-CbABF1Δ2 was co-transformed with the constructed *C. bungeana* cDNA library into *S. cerevisiae* strain AH109. Positive clones were identified based on growth on SD medium minus histidine, leucine, tryptophan, and adenine. The X-gal assay was performed to confirm the screening result. The inserted DNA fragments from the positive clones were sequenced and analyzed, and the full-length cDNA coding the candidate interaction protein was acquired by RACE as described above.

For validation of the yeast two-hybrid screening, coding regions of *CbABF1* and *CbSnRK2.6* were cloned into pDEST22 (pDEST22-CbABF1) as prey and pDEST32 (pDEST32-CbSnRK2.6) as bait, using the ProQuest^TM^ Two-Hybrid System with Gateway Technology (Invitrogen). The transformation of yeast and downstream analysis were carried out according to the manufacturer’s instructions.

### BiFC Assay

BiFC assays were carried out as described previously (Walter et al., 2004). Briefly, *CbABF1* and *CbSnRK2.6* coding regions were amplified and cloned into pUC-SPYNE and pUC-SPYCE, respectively. The resulting constructs (CbABF1-nYFP and CbSnRK2.6-cYFP) were transformed into *A. tumefaciens* strain GV3101 and infiltrated into the 4-week-old leaves of tobacco plants. YFP fluorescence was observed with a laser scanning confocal microscope (Leica TCS SP8).

### Dual Luciferase Reporter Assays

We synthesized a modified *RD29B* promoter contains five tandem copies of a 77-bp fragment of *RD29B* promoter and the minimal *RD29B* promoter as described (Yoshida et al., 2015), and the 77-bp fragment contains two ABRE *cis*-elements. The synthesized RD29B promoter was inserted into the transient expression vector pGreenII 0800-LUC to serve as a reporter plasmid (RD29B:LUC). The coding regions of CbABF1, CbABF1Δ2, and CbSnRK2.6 were amplified and cloned into the transient expression vector pGreenII 62-SK to serve as effect plasmids (pGreenII 62SK-CbABF1, pGreenII 62SK-CbABF1Δ2, and pGreenII 62SK-CbSnRK2.6, abbreviated as CbABF1, CbABF1Δ2, and CbSnRK2.6, respectively). The resulting plasmids were transformed into Agrobacterium (GV3101 with the pSoup-P19 helper plasmid). Four-week-old tobacco leaves were co-infiltrated with Agrobacterium harboring the above plasmids in the following combinations and ratios: RD29B: LUC (100%); RD29B: LUC + CbABF1 (1:9); RD29B: LUC + CbABF1Δ2 (1:9); and RD29B: LUC + CbABF1 + CbSnRK2.6 (1:4.5:4.5). After an additional incubation of 3 days, the activities of firefly luciferase under the control of the *RD29B* promoter (RD29B:LUC) and Renilla luciferase under the control of the 35S promoter (35S:REN) were assayed using the dual luciferase assay reagents (Promega, Madison, WI, United States) as described (Hellens et al., 2005; Hu et al., 2016). Normalized data (LUC/REN ratio) from 12 independent biological samples are presented.

### Transformation of Tobacco and Generation of Transgenic Plants

The *CbABF1* coding region was inserted into the PMDC32 vector (Curtis and Grossniklaus, 2003) using the LR recombination reaction with pDONR/zeo-CbABF1. The resulting *CbABF1* constructs driven by the CaMV35S promoter were delivered into tobacco leaves using the *A. tumefaciens* strains GV3101 for *Agrobacterium*-mediated leaf disk transformation to generate transgenic tobacco plants. Seeds from the transgenic plants were selected on MS medium with 50 mg L^-1^ of hygromycin. Two T3 homozygous lines (OE7 and OE17) were chosen for detailed analyses. Expression of *CbABF1* in transgenic lines was confirmed by RT-PCR using primers AQ-F and AQ-R (Supplementary Table S1), and *NtEF1*-α (*AF120093*) was used as an internal control (Schmidt and Delaney, 2010).

### Stress Treatments of Transgenic Tobacco Plants

Four-week-old plants growing in soil were used for freezing and drought tolerance test (80–100 plants for each test). For measurement of cold tolerance, freezing treatments were imposed as described previously (Novillo et al., 2004). Briefly, after subjecting 4-week-old plants to a freezing treatment at -4°C for 11 h, 18 plants were used to analyze ion leakage (IL), and the rest plants were incubated at 4°C for 1 day and then returned to 25°C. Survival rates were determined 5 days later. To impose drought, water was withheld from the plants. After 12 days of drought treatment, 18 plants were used to analyze IL, while the rest plants were re-watered and survival rates were determined 5 days after re-watering.

To measure the content of malonaldehyde (MDA), ROS, and antioxidant enzymes activities, 4-week-old WT and transgenic tobacco plants were put into a -2°C chamber for 8 h or withheld water for 8 days. Samples were collected and analyzed immediately or immersed rapidly in liquid nitrogen and stored at -80°C.

### Analysis of IL, MDA, ROS Contents, Water Loss, and Antioxidant Enzymes Activities

Ion leakage was quantified using previously described methods (Dong et al., 2006), from the detached leaves of the untreated and treated plants at the indicated time points. MDA contents were determined as described previously using the thiobarbituric acid (TBA) reaction as described previously (Ueda et al., 2015). Superoxide radicals (O_2_^-^) and hydrogen peroxide (H_2_O_2_) were detected by Superoxide Anion Assay Kit (Solarbio, China) and Micro Hydrogen Peroxide (H_2_O_2_) Assay Kit (Solarbio) according to the manufacturer’s instructions.

Water loss was quantified with 4-week-old plants grown in soil. Rosette leaves of identical size and stage were severed and fresh weight was weighed before and after exposed in air. The interval of each count was 10 min.

Activities of SOD (EC 1.15.1.1), CAT (EC 1.11.1.6), and POD (EC 1.11.1.7) were assayed using Total Superoxide Dismutase Assay Kit with WST-8 (Beyotime, China), Catalase Assay Kit (Beyotime), and Peroxidase Assay Kit (Solarbio), respectively, according to the manufacturers’ instructions. One unit of SOD is defined as the amount of enzyme needed to exhibit 50% dismutation of the superoxide radical, while one unit of CAT will decompose 1.0 μ mole of H_2_O_2_ per min at pH 7.0 at 25°C. For peroxidase assay, peroxidase activity was determined spectrophotometrically at 470 nm using guaiacol as a phenolic substrate with hydrogen peroxide, one unit of POD is defined as a change in absorbance of 0.01 min^-1^ in 1 mL of reaction mixture. All experiments were repeated at least three times and the average values are reported.

### Expression Analysis of Stress-Responsive Genes by qRT-PCR

Four-week-old tobacco plants were subjected to freezing (-2°C for 5 h) or drought (shoots were placed on filter papers to dry for 5 h). Tobacco leaves were collected, and qRT-PCR was performed as described above to evaluate the expression levels of six stress-responsive genes, including *NtLEA5* (AF053076), *NtERD10C* (AB049337.1), *NtLTP1* (X62395), *NtCAT1* (U93244.1), *NtSOD* (AB093097), and *NtPOX* (D42064.1) (Hu et al., 2013). *NtEF1*-α (AF120093) was used as an internal control for normalization (Schmidt and Delaney, 2010). For the analyzed genes, amplification efficiencies were between 0.9 and 1.1. The fold changes in the transcript levels are presented as the mean ± the standard deviation (SD) of three biological replicates using Pfaffl’s method (Pfaffl, 2001).

## Results

### Cloning and Sequence Analysis of *CbABF1*

**FIGURE 2:**
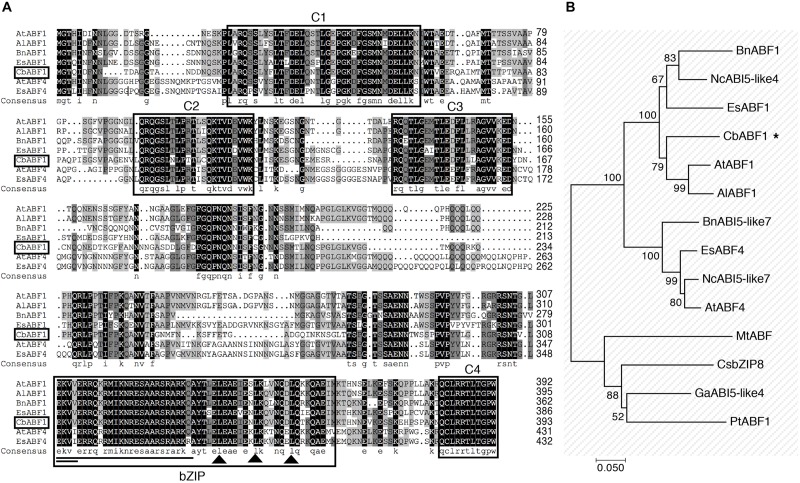
Sequence analysis of CbABF1. **(A)** Multiple sequence alignment of the deduced amino acid sequence of CbABF1 with its close orthologs. Compared proteins are from *Arabidopsis thaliana* (AtABF1, NP_564551.1; AtABF4, NP_566629.1), *Arabidopsis lyrata* subsp. *lyrata* (AlABF1, XP_002891529.1), *Brassica napus* (BnABF1, AGG35955.1), and *Eutrema salsugineum* (EsABF1, AFN84600.1; EsABF4, AFN84603.1). Black and gray shaded backgrounds indicate identical or similar amino acids, respectively. The basic region and the three heptad leucine repeats, two important bZIP signatures, are shown by a single line and black triangles, respectively. C1, C2, C3, and C4 are conserved regions containing residues of serine and threonine. A nuclear localization signal is highlighted by the double line. **(B)** Phylogenetic relationships between CbABF1 and its orthologs. The phylogenetic tree was constructed by the neighbor-joining method in MEGA 7.0. The numbers in the branches are the bootstrap values from 1000 replicates expressed as percentages. The sources of the CbABF1 orthologs and accession numbers are: BnABF1, *Brassica napus* (AGG35955.1), NcABI5-like4, *Noccaea caerulescens* (JAU90609.1), EsABF1, *Eutrema salsugineum* (AFN84600.1), CbABF1, *Chorispora bungeana* (MH213062), AtABF1, *Arabidopsis thaliana* (NP_564551.1), AlABF1, *Arabidopsis lyrata* (XP_002891529.1), BnABI5-like7, *Brassica napus* (NP_001303205.1), EsABF4, *Eutrema salsugineum* (AFN84603.1), NcABI5-like7, *Noccaea caerulescens* (JAU59323.1), AtABF4, *A. thaliana* (NP_566629.1), MtABF, *Medicago truncatula* (XP_003607958.1), CsbZIP8, *Camellia sinensis* (AGG39700.1), GaABI5-like4, *Gossypium arboreum* (KHG05529.1), and PtABF1, *Populus trichocarpa* (XP_006384405.1). CbABF1 was labeled by an asterisk.

An EST (GenBank: JK711564.1) with homology to ABF/AREB was found among those differentially expressed in response to freezing stress in *C. bungeana*. To isolate the corresponding full-length gene, we used the rapid amplification of cDNA ends (RACE) method. A 1482-bp cDNA containing a complete open reading frame of 393 amino acids was obtained (GenBank: MH213062). The deduced protein has a bZIP domain of 54 amino acids composed of a leucine zipper and a basic region, four conserved phosphorylation sites (C1–C4), and a putative nuclear localization signal (NLS) (Uno et al., 2000), but lacked a glutamine-rich domain and a casein kinase II phosphorylation site compare with ABF1 in *Arabidopsis* (Figure 2A). The protein showed high similarity with other ABF/AREB transcription factors from *A. thaliana*, *Arabidopsis lyrata*, *Eutrema salsugineum*, and *Brassica napus*. A phylogenetic analysis by neighbor-joining method showed that this protein was closely related to AtABF1, AlABF1, EsABF1, BnABF1, and NcABI5-Like4 (Figure 2B). Based on the analysis of conserved domains and phylogenetic comparisons with other ABF/AREB proteins, we concluded that CbABF1 belonged to the subfamily of AREB in the bZIP family (Jakoby et al., 2002), and designated it *C. bungeana ABF1* (*CbABF1*).

### *CbABF1* Expression Is Strongly Induced by Cold and Drought Stress

**FIGURE 3:**
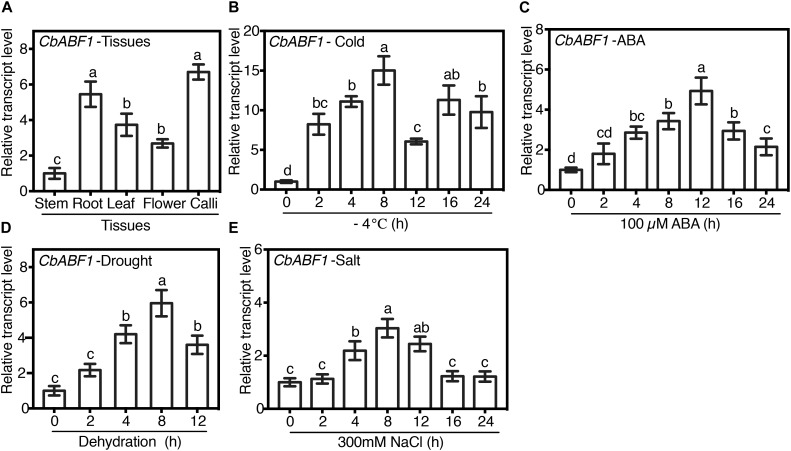
Expression profiles of *CbABF1*. **(A)** Expression of *CbABF1* in different tissues. The cDNAs for analysis were prepared from the regenerated plants and calli of *C. bungeana*. The stem value was defined as 1.0, and the expression levels in other tissues were normalized accordingly. **(B–E)** Expression profiles of *CbABF1* in response to abiotic stresses. Two-week old sub-cultured *Chorispora bungeana* seedlings were subjected to cold (–4°C), abscisic acid (ABA, 100 μM), or salt (NaCl, 250 mM) for 0–24 h, or drought (shoots were placed on filter paper) treatments for 0–12 h. For each assay, the expression level at time point 0 was defined as 1.0, and the expression levels at other time points were normalized accordingly. *C. bungeana ACTIN* (*CbACTIN*) was used as the internal control for qRT-PCR analysis. Error bars represent SD (*n* = 3). One-way ANOVA (Tukey test) was performed, and statistically significant differences are indicated by different lowercase letters (*P* < 0.01). Values shown are derived from experiments that were performed at least three times with similar results, and representative data from one repetition are presented.

To characterize the expression pattern of *CbABF1* in different tissues, the transcription of *CbABF1* in stem, root, leaf, flower, and callus of *C. bungeana* was examined by quantitative reverse-transcription PCR (qRT-PCR). Expression of *CbABF1* was detected in all screened tissues (Figure 3A). The highest expression was observed in the root and calli.

To explore the expression of *CbABF1* in response to various stressors and the phytohormone ABA, a time course experiment was conducted by qRT-PCR with regenerated seedlings of *C. bungeana*. We first examined the effects of cold stress. *CbABF1* was strongly induced by freezing stress and reached a maximum, 15-fold of the control, approximately 8 h after the freezing treatment started. Expression was substantially lower at 12 h, increased to around 12-fold of the control at 16 h and declined again by 24 h (Figure 3B). In the ABA treatment, *CbABF1* expression at 12 h reached its maximum level, around fivefold of the control, not as high as in the freezing treatment (Figure 3C). In the salt-stress treatment, *CbABF1* expression was only slightly induced by high salinity, with the maximum expression at 8 h (Figure 3D). In the drought-stress treatment, the expression of *CbABF1* began to increase at 2 h and reached its maximum level at 8 h, around sixfold of the control (Figure 3E). Overall, the results of the stress treatments suggest that *CbABF1* was most induced by cold, followed by drought.

### CbABF1 Is Located in the Nucleus

**FIGURE 4:**
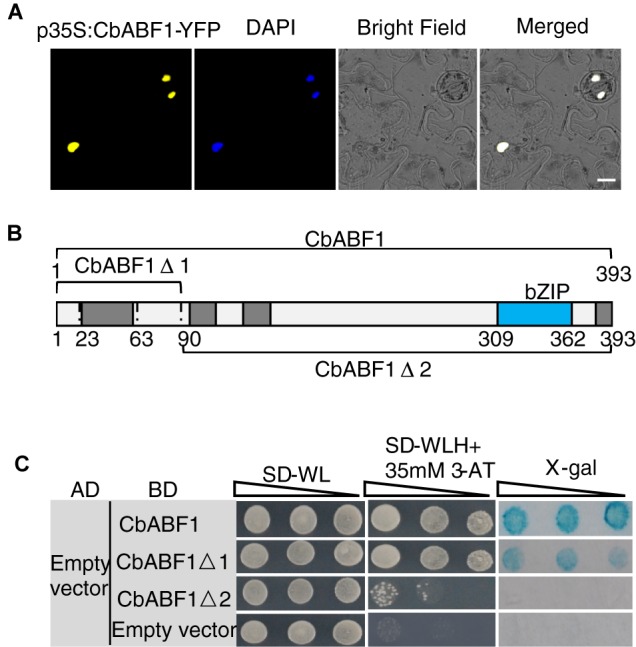
CbABF1 subcellular localization and transactivation activity assays. **(A)** Localization of p35S:CbABF1-YFP in tobacco leaves, DAPI staining was used to indicate the nuclei. Bars = 15 μm. **(B)** A schematic diagram of the CbABF1 protein sequence and its truncated fragments that were fused to the GAL4 DNA-binding domain expressed from the yeast vector pGBKT7. The bZIP domain and four conserved domains are indicated. **(C)** Transactivation analysis of CbABF1 in yeast. The constructs for expression of the GAL4 DNA-binding domain fused with CbABF1, CbABF1Δ1, and CbABF1Δ2 were co-transformed with empty pGADT7 into yeast strain AH109, respectively. A 10-fold dilution series (10^-1^ to 10^-3^) of transformants was spotted on SD medium without Leu and Trp (–LW), or without Leu, Trp, and His (–LWH) plus 35 mM 3-amino-1,2,4-triazole (3-AT). The plates were incubated at 30°C for 4 days and subjected to the X-gal assay. Empty pGBKT7 with empty pGADT7 was used as the negative control.

According to the sequence analysis, a putative NLS exists in the bZIP domain of CbABF1 protein, implying the possible localization of CbABF1 to the nucleus. To examine the subcellular localization of CbABF1, the *CbABF1* coding region without its native stop codon was fused in frame with the N-terminal of yellow fluorescent protein (YFP), under the control of the *Cauliflower mosaic virus* (CaMV) 35S promoter and NOS terminator. Tobacco (*N. tabacum*) leaves were transformed with this 35S:CbABF1-YFP fusion construct via *Agrobacterium*-mediated transformation. YFP fluorescence was observed exclusively in the nuclei of tobacco cells (Figure 4A), indicating that CbABF1 was located to the nucleus.

### CbABF1 Has Transactivation Activity That Is Dependent on the N-Terminal Region of 89 Residues

The nuclear localization of CbABF1 prompted us to examine whether CbABF1 has transactivation activity. CbABF1 was fused to GAL4 DNA Binding domain of vector pGBKT7 to generate pGBKT7-CbABF1, which was co-transformed with empty pGADT7 into yeast. Yeast cells transformed with empty pGBKT7 and pGADT7 were used as negative control. All transformants grew well on SD/-Trp/-Leu plates. However, when cultured on the SD/-Trp/-Leu-/-His plates that contained 35 mM 3-amino-1,2,4-triazole (3-AT), the transformants with pGBKT7-CbABF1 still grew well and turned blue in the X-gal assay (Figure 4), reflecting that CbABF1 has transactivation activity. To determine the activation domain in CbABF1, two truncated coding regions of CbABF1 were amplified and cloned to pGBKT7 to generate fusion plasmids pGBKT7-CbABF1Δ1 (Amino acid 1–89) and pGBKT7-CbABF1Δ2 (Amino acid 90–393) (Figure 4B), which were individually co-transformed with pGADT7 into yeast. When cultured on the SD/-Trp/-Leu/-His plates that contained 35 mM 3-AT, the transformants with pGBKT7-CbABF1Δ1 still grew well, whereas those with pGBKT7-CbABF1Δ2 and the negative control did not grow (Figure 4C). Further, the transformants with pGBKT7-CbABF1Δ1 turned blue during the X-gal assay. Together, our results indicated that CbABF1 had transactivation activity that depended on the presence of 89 residues of the N-terminal region.

### CbABF1 Interacts With a Snf1-Related Protein Kinase

**FIGURE 5:**
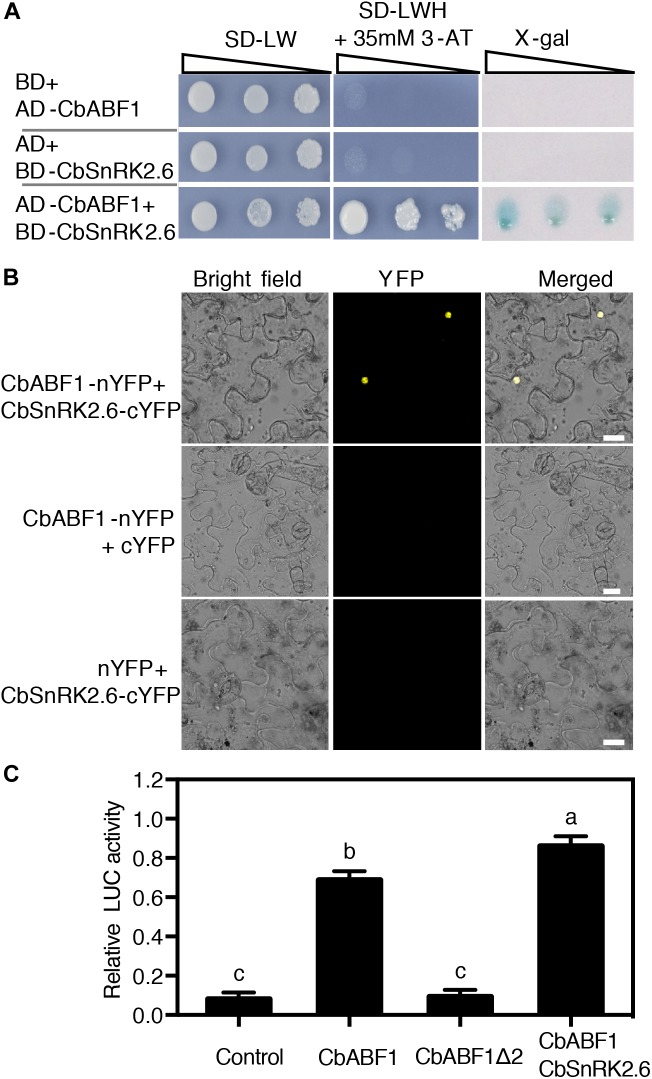
CbABF1 interacts with CbSnRK2.6. **(A)** CbABF1 interacts with CbSnRK2.6 as determined by yeast two-hybrid assays. Yeast strain Mav203 co-transformed with CbSnRK2.6-pDEST32 (bait) and CbABF1-pDEST22 (prey) was subjected to the X-gal assay. Mav203 cells co-transformed with CbSnRK2.6-pDEST32/pDEST22 (empty vector) or CbABF1-pDEST22/pDEST32 (empty vector) were used as negative controls. Ten-fold dilution series (10^-1^ to 10^-3^) of transformants were spotted on SD medium –LW, or SD medium –LWH + 3-AT. The plates were incubated at 30°C for 4 days and subjected to the X-gal assay. **(B)** CbABF1 interactions with CbSnRK2.6 *in vivo* as determined by bimolecular fluorescence complementation (BiFC) assays in tobacco leaf epidermal cells. Bars = 20 μm. Yellow fluorescent protein was detected at an approximate frequency of 3.04% (86 out of 2,830 tobacco leaf epidermal cells analyzed exhibited BiFC events). **(C)** Relative luciferase activity from the dual luciferase reporter assays of *RD29B* promoter containing ABRE elements in tobacco leaves. CbABF1Δ2 is the C-terminal fragment mentioned in Figure 4. Error bars represent SD (*n* = 12). One-way ANOVA (Tukey–Kramer test) was performed, and statistically significant differences are indicated by different lowercase letters (*P* < 0.01). Values shown are derived from experiments that were performed three times with similar results, and representative data from one repetition are presented.

To identify proteins that interact with CbABF1, we performed yeast two-hybrid assays. We constructed a *C. bungeana* cDNA library fused to the yeast GAL4 activation domain. A CbABF1 fragment lacking the 89 residues of the N-terminal region (CbABF1Δ2) fused to GAL4 DNA binding domain was used as bait. Approximately 3 million yeast transformants were screened, and 12 positive clones were isolated. Four of these clones encode the same peptide (Sequence in Supplementary Figure S1) with similarity to C-terminal part of *Arabidopsis* SnRK2.6/OST1, and we selected this protein for further analysis. We cloned the corresponding full-length gene from *C. bungeana* by RACE and found that it shares high amino acid identity and similarity to *Arabidopsis* SnRK2.6/OST1, a Snf1-related protein kinase. Consequently, we named it *CbSnRK2.6* (*C. bungeana SnRK2.6*, MH213063). We confirmed the interaction of full-length CbSnRK2.6 with CbABF1 in yeast cells (Figure 5A). In addition, BiFC analysis using the epidermal cells of tobacco leaves confirmed that CbABF1 interacted with CbSnRK2.6 in plant cells (Figure 5B). Hence, CbABF1 interacts with a Snf1-related protein kinase, *CbSnRK2.6*.

### CbSnRK2.6 Enhances the Transactivation of CbABF1 on Stress Responsive Genes Containing ABRE *cis*-Element in Plant Cell

We performed a dual luciferase reporter assay in tobacco leaves to examine the effect of CbABF1 on ABRE *cis*-element containing promoter *in vivo*. Since a 77-bp fragment containing two ABRE elements of *RD29B* promoter were usually used to identify ABRE binding proteins (Yoshida et al., 2015), we synthesized a *RD29B* promoter containing five tandem copies of the 77-bp fragment and the minimal *RD29B* promoter (Fujita et al., 2005). The synthesized *RD29B* promoter was inserted into the transient expression vector pGreenII 0800-LUC to serve as a reporter plasmid (RD29B:LUC). The coding regions of CbABF1, CbABF1Δ2, and CbSnRK2.6 were amplified and cloned into the transient expression vector pGreenII 62-SK to serve as effecters. Expectedly, CbABF1 recognized the ABRE *cis*-element which resulted in transactivation of the reporter gene. While deleted the N-terminal 89 amino acids of CbABF1, CbABF1Δ2 failed to activate the reporter gene, which is consistent with the transactivation assay in yeast. Furthermore, CbSnRK2.6 enhanced the transactivation of CbABF1 on the *RD29B* promoter (Figure 5C).

### CbABF1 Confers Freezing and Drought Tolerance in Transgenic Tobacco Plants

**FIGURE 6:**
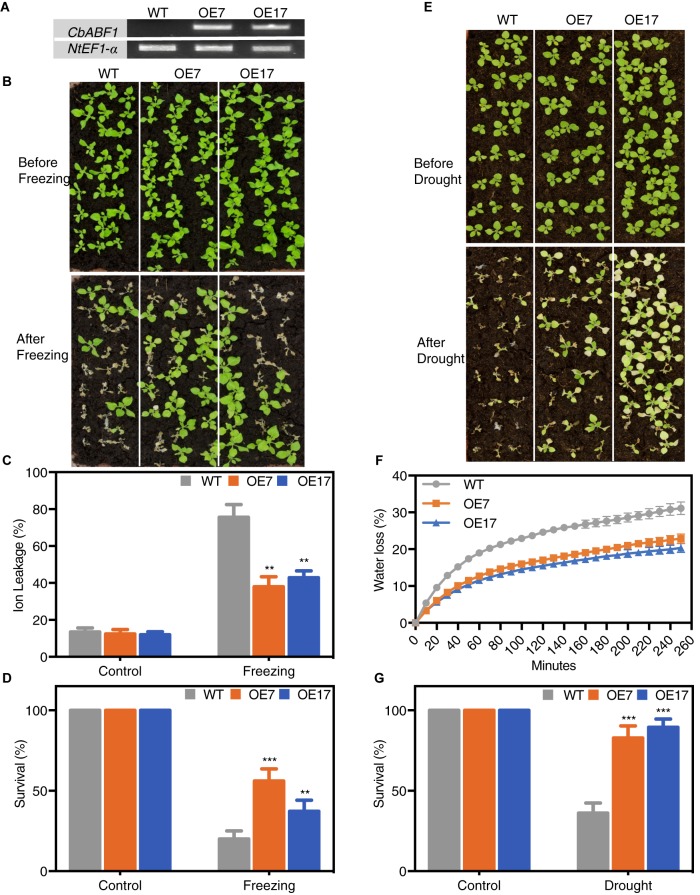
Analysis of freezing and drought tolerance in *35S:CbABF1* transgenic plants. **(A)** Expression analysis of the *CbABF1* gene in transgenic tobacco lines. The wild type (WT) and transgenic lines (OE7 and OE17) were cultured on MS medium for 2 weeks and whole seedlings were used to extract RNA to detect gene expression using *NtEF1*-α as an internal control. **(B)** Four-week-old wild type (WT) and transgenic tobacco lines OE7 and OE17 (*35S:CbABF1*) were subjected to freezing for 11 h at –4°C. After incubation at 4°C for 1 day, plants were returned to 25°C. Survival rates were determined, and photographs were taken 5 days later. **(C,D)** Ion leakage and survival rates of WT and transgenic lines after freezing treatment in panel **(B)**. **(E)** Four-week-old wild type (WT) and transgenic tobacco lines OE7 and OE17 (*35S:CbABF1*) were drought stressed by withholding water for 12 days, and then re-watered for 5 days before photographs were taken. **(F)** Water loss from the leaves of wild type (WT) and transgenic tobacco lines OE7 and OE17 at various time points after detachment of leaves. **(G)** Survival rates of WT and transgenic lines after drought treatment in panel **(E)**. Error bars represent SD [*n* = 18 in panels **(C,F)**; *n* = 72 in panels **(D,G)**]. Two-way ANOVA (Tukey test) was performed, and statistically significant differences are indicated by asterisks (^∗∗^*P* < 0.01 and ^∗∗∗^*P* < 0.001). Values shown are derived from experiments that were performed at least three times with similar results, and representative data from one repetition are presented.

To investigate the function of *CbABF1* in response to abiotic stress, transgenic tobacco plants expressing *CbABF1* driven by the CaMV 35S promoter were generated by *Agrobacterium*-mediated transformation of tobacco leaf disks, and we eventually obtained five T3 homozygous lines. Expression of *CbABF1* in two representative lines (OE7 and OE17) was verified by RT-PCR analysis (Figure 6A). No obvious morphological differences were observed between the transgenic lines and wild-type (WT) plants (Figure 6B).

After freezing treatment (-4°C for 11 h), almost all WT plants collapsed with leaves showing dark green, whereas majority of the *35S:CbABF1* plants kept their initial status. Further, IL was relatively lower in the transgenic plants compared with the WT (Figure 6C). Thus, cells in WT plants were more damaged by freezing stress than were those of *35S:CbABF1* plants. After 5 days of recovery, both *35S:CbABF1* transgenic lines (OE7 and OE17) had high survival rates (58 and 38%, respectively), whereas WT plant survival rate was <20% (Figure 6D). Which indicated that over-expression of *CbABF1* enhanced freezing stress tolerance in transgenic tobacco plants.

Because endogenous *CbABF1* was up-regulated by drought stress as well as cold in *C. bungeana*, we examined the drought-stress tolerance of the *35S:CbABF1* tobacco lines compared with WT tobacco plants. After water was withheld for 12 days, all plants exhibited reduced growth and increased chlorosis. However, the WT plants were much more withered and chlorotic, and collapsed more than the transgenic lines (Figure 6E). After 5 days of re-watering, both transgenic lines showed high survival rates (90% for OE7; 85% for OE17). By contrast, only 32% of the WT plants survived (Figure 6G). Consistent with the visible phenotypes, the transgenic plant leaves showed significantly lower water loss and IL rates, compared with wild-type leaves (Figure 6F and Supplementary Figure S2). These results indicated that over-expression of *CbABF1* also enhanced drought tolerance in transgenic plants.

### Plants Expressing CbABF1 Have Reduced ROS Levels and Less Oxidative Damage

**FIGURE 7:**
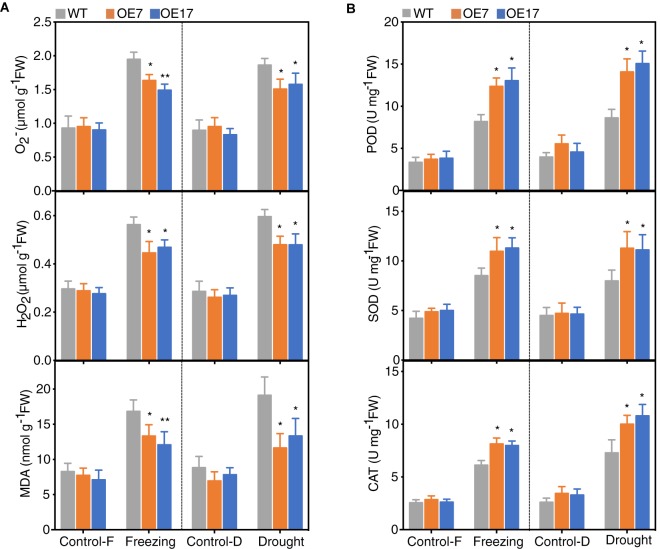
Analysis of reactive oxygen species (ROS) accumulation and scavenging systems. **(A)** Analysis of reactive oxygen species (ROS) accumulation and malondialdehyde (MDA) content in WT and transgenic lines under freezing and drought conditions. **(B)** Activities of peroxidase (POD), superoxide dismutase (SOD), and catalase (CAT) in WT and transgenic lines under freezing and drought conditions. Four-week-old tobacco plants were subjected to freezing (–2°C for 8 h) and drought (withholding water for 8 days) stress. Tobacco leaves were collected for analysis. Control-F, control in freezing treatment, Control-D, control in drought treatment. Data are means (±SD) calculated from three replicates. Asterisks indicate significant difference between the WT and the two transgenic lines (^∗^*P* < 0.05, ^∗∗^*P* < 0.01, by two-way ANOVA Tukey test). Three biological experiments were performed, which produced similar results.

Given that stress conditions generate ROS that causes oxidative damage, and the expression of *CbABF1* enhanced freezing and drought tolerance in the transgenic plants, we tested whether CbABF1 might regulate ROS levels in response to stresses. We examined two prominent ROS species, H_2_O_2_ and O^2-^, in the *35S:CbABF1* and WT plants. Both H_2_O_2_ and O^2-^ contents increased in the freezing and drought-stress treatments for all plants; however, the accumulation of H_2_O_2_ and O^2-^ in the transgenic plants was lower than in WT plants (Figure 7A).

To further quantify oxidative damage, we measured malondialdehyde (MDA) as a marker for lipid peroxidation and found that MDA content was significantly lower in the *35S:CbABF1* plants than in WT plants. As the ROS-scavenging enzymes superoxide (SOD), peroxidase (POD) and catalase (CAT) play crucial roles in detoxifying ROS. Thus, the activities of these enzymes were measured in plants under control and stress conditions. After freezing and drought stress treatment, the activities of POD, SOD, and CAT were significantly higher in the two transgenic lines (around twofold increased) compared to that of the WT plants (Figure 7B). Overall, these results demonstrated that heterologous expression of *CbABF1* could decrease stress-related ROS levels and alleviate oxidative damage by enhancing the activity of ROS-scavenging enzymes.

### Heterologous Expression of CbABF1 Enhances the Expression of Stress-Responsive Genes

**FIGURE 8:**
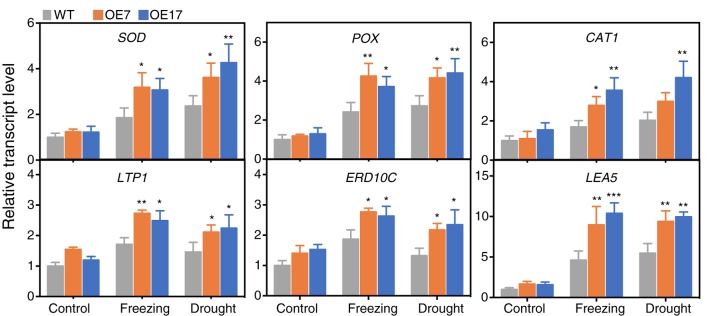
Expression levels of stress-responsive genes in wild type (WT) and *35S:CbABF1* transgenic plants under freezing and drought conditions. Four-week-old wild-type and transgenic (OE7 and OE17) tobacco plants were subjected to cold (–2°C for 5 h) and drought (shoots were placed on filter papers and allowed to dry for 5 h). Tobacco leaves were collected to detect expression levels of the genes via qRT-PCR. *NtEF1*-α (AF120093) was used as the internal control. Data represent mean values (±SD) from three independent replicates. Asterisks indicate statistical significance between the transgenic and WT plants (^∗^*P* < 0.05, ^∗∗^*P* < 0.01, ^∗∗∗^*P* < 0.001, two-way ANOVA Tukey test). Three biological experiments were performed, which produced similar results.

To acquire further insights into the transcriptional regulatory roles of *CbABF1*, the expression of down-stream stress-responsive genes and ROS-scavenging genes in the transgenic and WT plants were examined. The analyzed genes include *NtSOD*, *NtPOX*, and *NtCAT1* involved in ROS detoxification, *NtLEA5*, *NtERD10C* related to stress defense, and lipid-transfer protein genes *NtLTP1* (Hu et al., 2013). In the absence of stress, the expression of *NtSOD*, *NtPOX*, *NtCAT1*, *NtLEA5*, *NtERD10C*, and *NtLTP1* showed no significant difference between the transgenic and WT plants (Figure 8). When the plants were subjected to drought or freezing, all six genes were up-regulated in all plants. Notably, the expression levels of these six genes in the *35S:CbABF1* plants were significantly higher than that in the WT plants. These results indicated that expression of *CbABF1* in tobacco could enhance the expression of endogenous stress-related genes in response to freezing or drought stress.

## Discussion

ABFs/AREBs play significant roles in regulating stress-related genes in plant response to various adverse environment (Yoshida et al., 2015; Fernando et al., 2018; Wang et al., 2019). Although a number of ABFs/AREBs have been identified from different plant species in the last two decades (Uno et al., 2000; Johnson et al., 2008; Amir Hossain et al., 2010; Kerr et al., 2018), little is known about the roles of ABF/AREB transcription factors in alpine subnival plants, which suffer extremely environment. In this study, we cloned and characterized *CbABF1*, an ABF-like transcription factor from a representative alpine subnival plant, *C. bungeana*. Our findings have shown that CbABF1 could transactivate stress-related genes by binding ABRE *cis*-element in their promoters in response to cold, drought and abscisic acid, which was regulated by CbSnRK2.6. In addition, over-expression of *CbABF1* greatly enhanced freezing and drought tolerance in the transgenic plants, suggesting a potential role for *CbABF1* in molecular breeding.

Plant ABFs are induced by various abiotic stressors (Amir Hossain et al., 2010; Orellana et al., 2010; Wang et al., 2019). In *Arabidopsis*, the ABF members might have specific roles in response to different stresses, in addition to exhibiting functional redundancy. For example, *AtABF1* is mainly induced by cold, and its expression is lower than *ABF2*/*AREB1*, *ABF3*, and *ABF4*/*AREB2* in response to dehydration, high salinity and ABA treatment (Yoshida et al., 2015), implying that the primary role of ABF1 in *Arabidopsis* is in cold stress responses and related gene regulation. In this study, we found that the expression of *CbABF1* was strongly induced by cold, and also induced by drought, high salinity and ABA, but the accumulation of *CbABF1* transcript under salt and ABA treatments was lower than that in cold stress, similar to *AtABF1*. Considering that *CbABF1* rapidly and dramatically responds to freezing stress, we speculated that there may be additional cold-responsive *cis*-elements contained in the promoter. Indeed, promoter analysis by PlantCARE showed that among the stress-related *cis*-acting elements presented in the 1.5-kb promoter region (Supplementary Figure S3), a cold responsive *cis*-element, LTR, was exclusively presented in the promoter of *CbABF1* compared with *AtABF1* promoter (Lescot et al., 2002; Liu et al., 2016), suggesting that *CbABF1* may play an important role in improving cold tolerance in *C. bungeana*.

We found that CbABF1 interacted with a putative protein kinase CbSnRK2.6, which is most closely related to the *Arabidopsis* SnRK2.6/OST1. In *Arabidopsis*, SnRK2 protein kinases activate the ABF/AREB transcription factors directly by phosphorylation (Sirichandra et al., 2010; Ding et al., 2015). In *C. bungeana*, CbSnRK2.6 enhances the transactivation of CbABF1 on the ABRE *cis*-element. It is likely to regulate *CbABF1* in the similar way with AtSnRK2.6, which would suggest that the ABA-dependent pathway conserved in the alpine subnival plant that survives in extremely stressful conditions. However, how many Snf1-related protein kinases exist and how they function in *C. bungeana* still need further elucidation.

To elucidate potential roles for *CbABF1* in cold and drought tolerance, we characterized transgenic tobacco plants expressing *CbABF1*. The two selected transgenic lines had better survival rates in freezing and drought treatments compared with WT plants. Transgenic plants also had lower IL than WT under stress treatments, indicating that expression of *CbABF1* conferred tolerance to both freezing and drought stress. Importantly, the transgenic lines expressing *CbABF1* did not show growth retardation, suggesting that *CbABF1* may be a candidate genetic resource for engineering more stress-tolerant crops.

It is well documented that ROS can act as intracellular messengers (Chinnusamy et al., 2010; Liu et al., 2010); however, over-accumulation of ROS causes lipid peroxidation, resulting in MDA production in damaged plants. Therefore, MDA levels can report the damage to cellular components (Huang et al., 2010; Miller et al., 2010; Zhang et al., 2011). In our study, the MDA content of both transgenic tobacco lines was less than in WT plants in the freezing and drought treatments, suggesting that less toxic material was produced in the transgenic lines. Indeed, *CbABF1* expression remarkably reduced the accumulation of O^2-^ and H_2_O_2_, indicating that the scavenging systems in the transgenic plants might be more robust than in WT plants.

In ROS-scavenging systems, antioxidant enzymes play key roles. SOD catalyzes the dismutation of O^2-^ to oxygen and H_2_O_2_, which is then scavenged by CAT and POD (Miller et al., 2010). We found that activities of SOD, POD, and CAT in both transgenic lines were significantly higher than that of WT plants under freezing and drought stress. These results imply that *CbABF1* expression indeed enhanced antioxidant defense systems to eliminate ROS, which is in accordance with the reduction of ROS levels. It is noteworthy that the ROS-scavenging antioxidant-defense genes such as *Manganese Superoxide Dismutase* (*SOD*), *Catalase 1* (*CAT1*), and *Cationic Peroxidase* (*POX*) were up-regulated in the freezing and drought treatments, implying that the ROS-scavenging systems may be regulated at the transcription level. These results indicate that *CbABF1* is involved in the modulation of ROS accumulation by regulating the expression of antioxidant defense genes and thereby enhances plant tolerance to freezing and drought stress.

In summary, CbABF1, a transcription factor from the subnival plant *C. bungeana*, plays important roles in response to freezing and drought stress. CbABF1 transactivates the stress responsive genes by recognizing the ABRE *cis*-element, and the transactivation activity of CbABF1 depends on the N-terminal region that contains 89 residues. Through interacting with CbSnRK2.6, a Snf1-related protein kinase, CbABF1 may also be activated by phosphorylation, a process that has been well documented for AtABF1 in *Arabidopsis*. However, the specific mechanisms of stress tolerance in *C. bungeana* need further investigation. Our work shows that *CbABF1* greatly enhanced freezing and drought tolerance of transgenic tobacco plants, suggesting that *CbABF1* could be a candidate gene with which improve stress tolerance in crops.

## Author Contributions

LA and XY planned and designed the research and wrote the manuscript. XY, GZ, ZeZ, JY, XP, MS, YZ, ZW, GM, and ZiZ conducted the experiments. XY and YS analyzed the data.

## Conflict of Interest Statement

The authors declare that the research was conducted in the absence of any commercial or financial relationships that could be construed as a potential conflict of interest.
