# Supplementary material for: A Cryophyte Transcription Factor, CbABF1, Confers Freezing, and Drought Tolerance in Tobacco
Source: Front Plant Sci. 2019 May 31;10:699. doi: 10.3389/fpls.2019.00699 (PMC6555190; doi:10.3389/fpls.2019.00699)
Supplement: Supplementary file 1 [file Data_Sheet_1.PDF]

Supplementary Material

1 Supplementary Figures and Tables

1.1 Supplementary Figures

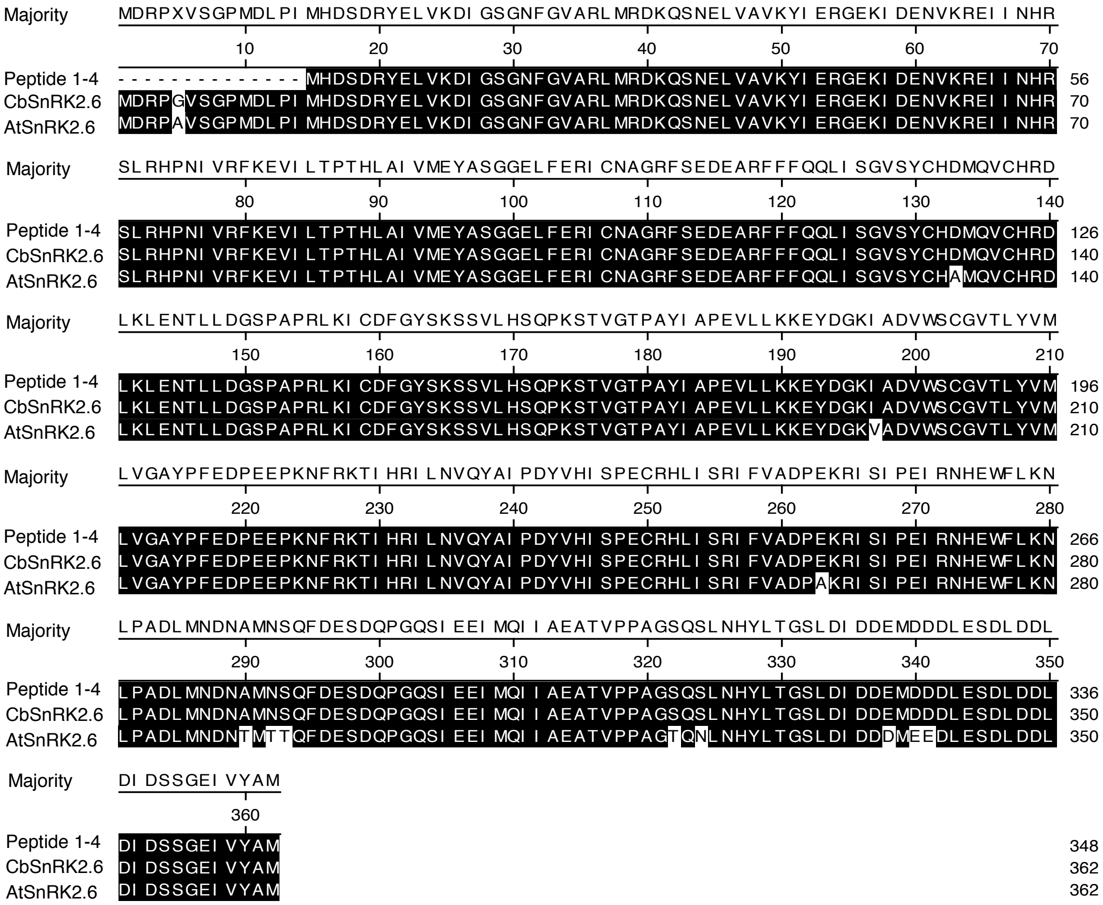

FIGURE S1 | Alignment of the amino acid sequences of Peptide1-4, CbSnRK2.6, and AtSnRK2.6. Peptide1-4, the four positive clones by yeast two hybrid; CbSnRK2.6, *Chorispora bungeana* SnRK2.6/OST1 (QBA17182.1); AtSnRK2.6, *Arabidopsis thaliana* SnRK2.6/OST1 (NP_567945.1).

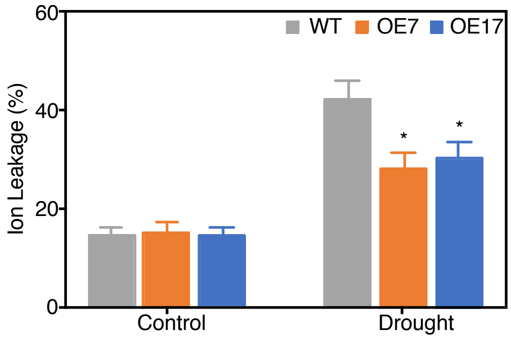

FIGURE S2 | Ion leakage of WT and transgenic lines after drought stress. Four-week-old wild type (WT) and transgenic tobacco lines OE7 and OE17 (*35S:CbABF1*) were drought stressed by withholding water for 12 d, and the leaves were detached for ion leakage assay. Error bars represent SD (n = 18). Two-way ANOVA (Tukey test) was performed, and statistically significant differences are indicated by asterisks (* $P < 0.05$). Values shown are derived from experiments that were performed three times with similar results, and representative data from one repetition are presented.

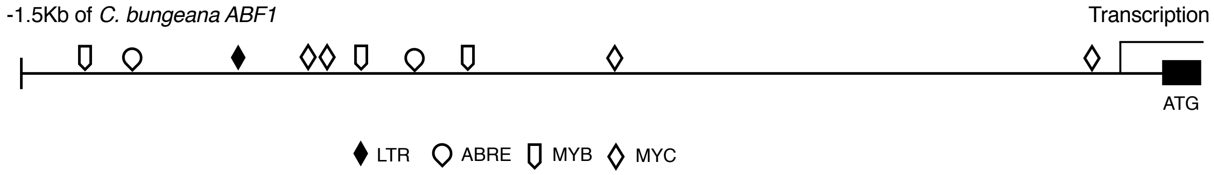

FIGURE S3 | Distribution of major stress-related *cis*-elements in the promoter region of *CbABF1*.

1.2 Supplementary Tables

TABLE S1 | List of primers used in present study.

Genes	Purpose	Primers	Sequences (5'-3')
CbABF1	5' RACE	5TR	AGGCGATGGATGCTCCTTCGAC
	3' RACE	3TR	AGGCTGAAATTATGAAAACCCATAATAACGAGG
CbABF1	q-PCR	AQ-F	AACACATTAGGTGAGCCAGG
		AQ-R	AGCAGGAACAGAACCACTAATAG
CbABF1	Full-length *ABF1* amplification	YH1	AATGCATATGATGGGTACTCAAATCGACTTCAACAACAC
		YH393	TCCAGTCGACTTACCAAGGACCCGTAAGCGTTC

CbABF1	Truncated *ABF1Δ1* amplification	YH1	AATGCATATGATGGGTACTCAAATCGACTTCAACAACAC
		YH89	TCCAGTCGACACTAATAGGTTGTGCTGGGGCTGC
CbABF1	Truncated *ABF1Δ2* amplification	YH90	AATGCATATGGGTTCTGTTCCTGCTGGTGAG
		YH393	TCCAGTCGACTTACCAAGGACCCGTAAGCGTTC
CbABF1	To construct *ABF1Δ2* effector	DL90	CGGGATCCGGTTCTGTTCCTGCTGGTGAG
		YH393	TCCAGTCGACTTACCAAGGACCCGTAAGCGTTC
CbABF1	To construct *ABF1* effector	DL1	CGGGATCCATGGGTACTCAAATCGACTTCAACAACAC
		YH393	TCCAGTCGACTTACCAAGGACCCGTAAGCGTTC
CbSnRK2.6	To construct *CbSnRK2.6* effector	DLS1	CGGGATCCATGGATCGGCCAGGAGTGAGTGGTC
		DLS362	TCCAGTCGACTCACATTGCATACACAATCTCTCC
CbABF1		PD-F	GGGGACAAGTTTGTACAAAAAAGCAGGCTTCATGGGTACTCAAATCGACTTCAAC
	Gateway	PD-R	GGGGACCACTTTGTACAAGAAAGCTGGGTCCCAGGACCCGTAAGCGTTC
CbACTIN	qRT-PCR	AC-F	GGATGCTTACGTTGGTGACGA
		AC-R	TCTCCATGTCATCCCAGTTGC
CbSnRK2.6	3' RACE	3TR	TTCTAAGTCTTCGGTGTTACATTCGC
	5' RACE	5TR	TAACATACAGAGTTACCCCACAAGACC
NtLEA5	qRT-PCR	LE-F	TTGAATCTGGGGTTTTGGTT
		LE-R	GGAAGCATTGACGAGCTAGG
NtERD10C	qRT-PCR	ER-F	AACGTGGAGGCTACAGATCG
		ER-R	GTTCCTCTTGGGCATGAGTT
NtLTP1	qRT-PCR	LT-F	GCAGAAGCCATAACCTGTGG
		LT-R	CAGTGGAAGGGCTGATCTTG
NtPOX	qRT-PCR	POX-F	TTAGCTGCTCGTGATGCTGT
		POX-R	AGTTGAGCTCTGAGGGTCCA
NtSOD	qRT-PCR	SOD-F	CTCCTACCGTCGCCAAAT
		SOD-R	GCCCAACCAAGAGAACCC
NtCAT1	qRT-PCR	CAT-F	AGCCGGTGGGAAGATTAGTT
		CAT-R	AAGCAAGCTTTTGACCCAGA
NtEF1-α	qRT-PCR	EF1-F	TGAGATGCACCACGAAGCTC
		EF1-R	CCAACATTGTCACCAGGAAGTG

Note: The underlined fragments are attB1 and attB2 sequences according to the gateway system.
